# Building on the 2025 national academies pharmacy workshop: ensuring access to community pharmacist care

**DOI:** 10.1093/haschl/qxag252

**Published:** 2026-09-18

**Authors:** Jennifer L Bacci, Stuart Beatty, John G Gums, Patricia H Fabel, Kyle Melin

**Affiliations:** Center for Pharmacy Practice Transformation, University of Washington School of Pharmacy, Seattle, WA 98195, United States; College of Pharmacy, Ohio Northern University, Ada, OH 45810, United States; College of Pharmacy, University of Florida, Gainesville, FL 32610, United States; Kennedy Pharmacy Innovation Center, University of South Carolina College of Pharmacy, Columbia, SC 29208, United States; School of Pharmacy, University of Puerto Rico, Suan Juan, Puerto Rico 00921

**Keywords:** community pharmacy services, health insurance reimbursement, reimbursement mechanisms, healthy policy

## Abstract

Growing medication use, chronic disease burden, and primary care provider shortages have intensified the need for additional models of care delivery in the United States. Community pharmacists are uniquely positioned to help address these challenges through medication management, chronic disease management, and timely access to care. Evidence demonstrates that pharmacist services improve medication use and therapeutic outcomes while lowering overall health care costs. Despite these benefits, pharmacists remain underutilized as patient care providers, largely due to inconsistent reimbursement and business models that continue to prioritize product dispensing over patient care. Building on the 2025 National Academies of Sciences, Engineering, and Medicine workshop, *Innovations in Pharmacy Training and Practice to Advance Patient Care*, this paper examines existing and proposed state and federal payment policies and how their design influences implementation and scalability in community pharmacy practice. Recommendations include broad medical benefit reimbursement across payers, broad patient and service eligibility, and deference to state-defined scope of practice. Additional reforms are needed to address implementation barriers related to electronic health record access and credentialing. Together, these reforms can support sustainable, scalable community pharmacist-provided care and improve care access, quality, and affordability.

Key points

- Community pharmacists are well positioned to address growing needs for medication management, chronic disease management, and timely access to care.
- State and federal payment policy design influences whether community pharmacist-provided care can be consistently implemented and scaled.
- Sustainable, scalable community pharmacist-provided care requires broad medical-benefit coverage across payers, broad patient and service eligibility, and deference to state-authorized scope of practice.

## Background

In May 2025, the National Academies of Sciences, Engineering, and Medicine (NASEM) hosted a two-day workshop focused on the profession of pharmacy called “Innovations in Pharmacy Training and Practice to Advance Patient Care.”^1^ One of the 4 key topic areas discussed was the financial sustainability of pharmacist-provided care, including existing and proposed state and federal policies in the United States (US) that promote financially supported patient care roles for pharmacists. In this policy analysis, we build on the discussion initiated at the NASEM workshop by examining existing and proposed state and federal policies supporting payment for community pharmacist-provided care and identifying reforms needed to enable sustainable, scalable implementation. This analysis extends policy discussions by focusing on payment for pharmacist-provided care through the medical benefit, rather than scope of practice (the patient care functions pharmacists are authorized to perform), and by considering how policy design influences implementation and scalability in community pharmacy practice.

### Needs of patients and communities

Patients and communities in the US face growing needs for medication management, chronic disease management, and timely access to care.^2-6^ These needs are driven by increasing medication use and chronic disease burden, persistent medication-related morbidity and mortality, and growing shortages in primary care, particularly among older adults and rural and underserved communities.^7-10^

Medication use continues to increase, particularly among older adults, creating a growing need for effective medication management.^6^ According to the National Health and Nutrition Examination Survey, the proportion of the population taking 5 or more prescription medications has been increasing, reaching 5.1% between 2017 and 2020. Among people 65 years of age and older, this number rises to 43%, up from 33% in 2004. An estimated $528 billion is spent annually in the US on medication-related morbidity and mortality, driven by nonadherence, delayed evidence-based treatment practice, antibiotic misuse, medication errors, suboptimal generic use, and mismanaged polypharmacy.^9^ Medication nonadherence alone is associated with approximately 125 000 deaths, 10% of hospitalizations per year, and $100 billion in health care costs per year.^11^ These data highlight that medication-related problems are not simply isolated issues of prescribing or adherence, but a major contributor to preventable morbidity, mortality, and healthcare spending, reinforcing the need for care focused on ensuring medications are used safely and effectively.

At the same time, chronic disease continues to place substantial demands on the health care system. Diabetes, hypertension, and high cholesterol increase the likelihood of developing heart disease, the leading cause of death in the US.^12^ People living in rural areas, 14% of the US population, are more likely to develop complications or die from chronic illnesses such as diabetes and heart disease compared to those living in metropolitan areas.^13^ Rates of morbidity and mortality are especially high in the rural South.^14^ These disparities are further complicated by the shortage of primary care providers. A 2024 National Center for Health Workforce Analysis report found there are 7501 primary care health professional shortage areas (HPSAs) in the US.^10^ Without changes to primary care delivery, the US may face a shortage of over 87 150 full-time equivalent primary care providers by 2037 representing a 27% shortage, largely in rural areas.

Primary care shortages affect not only the effective management of chronic disease but also timely access to care for acute health needs. In 2022, 10.6% of US adults reported being unable to find an available primary care appointment when needed.^15^ Another national study found that patients seeking primary care appointments waited an average of 28.5 days.^16^ Delays in care can lead to worsening health outcomes and increased healthcare costs.^17,18^ When a patient cannot access primary care when they need it, they may instead turn to emergency departments or urgent care for conditions that could be managed in a lower-cost setting.

Together, increasing medication-related needs and chronic disease burden, coupled with primary care shortages and delayed access to care, create a need for greater capacity across the health care system. Additional models of care are needed to expand access and complement existing primary care infrastructure, particularly in communities where access to care is already limited.

### Meeting the needs through community pharmacy

Community pharmacists are well positioned to help address growing needs for medication management, chronic disease management, and timely access to care. Community pharmacies have been an integral part of the US health care system for over 200 years.^19^ During the 20^th^ century, pharmacy practice evolved from compounding prescriptions to dispensing manufactured medication products. In 2000, the Doctor of Pharmacy degree became the entry-level credential for pharmacists, producing more clinically trained medication experts. Around the same time, state pharmacy practice acts began expanding pharmacists' scope of practice to include the provision of health-related services, such as immunizations, chronic disease management, wellness and prevention screenings, test-and-treat programs, and patient education and counseling.^19,20^

Community pharmacists are highly accessible health care providers, well positioned to provide timely and meaningful care to patients. Patients visit their pharmacy more frequently than their primary care provider, with visit frequency estimated to be 1.5 to 10 times higher.^21-23^ This frequent contact enables community pharmacists to identify and address medication-related problems, manage chronic disease and non-emergency acute illness, reinforce treatment plans, and provide preventative care throughout a patient's care journey rather than during intermittent healthcare encounters.

Evidence demonstrates that community pharmacists can extend primary care capacity through the provision of medication management and chronic disease management in the context of team-based care. Systematic reviews and meta-analyses of community pharmacist-led interventions have found improvements in clinical outcomes, including blood pressure, glycemic control, and lipid management, and suggest potential cost savings without increasing health utilization.^24-28^ Evidence also supports integrating pharmacists into primary care teams, where such models have been associated with improvements in medication management, prescribing, health care utilization, and clinical outcomes.^24,29-32^ By providing ongoing medication management, monitoring, and follow-up in collaboration with other health care providers, community pharmacists may complement existing primary care and expand the capacity of the care team.

Community pharmacists may also help meet patients' needs for timely access to care for non-emergency acute conditions. In 2023, approximately 60% of Americans indicated they are likely to visit a community pharmacy first when experiencing non-emergency medical issues.^33^ Following the COVID-19 pandemic, community pharmacists are increasingly involved in addressing acute conditions through services such as testing, treatment, and prescribing. In a three-year study comparing community pharmacist-provided care for non-emergency acute issues with care delivered in primary care, urgent care, and emergency department settings, community pharmacist care was associated with substantially lower costs while maintaining comparable quality.^34^ Across 46 pharmacies and 175 pharmacists, median costs of care were nearly $280 lower than traditional care settings with noninferior return visit rates. These data suggest a growing role of community pharmacists in addressing care gaps in non-emergency acute conditions while helping alleviate pressure across the broader healthcare system.

Together, community pharmacists' clinical training, accessibility, and demonstrated ability to provide effective care position community pharmacy as an important setting for expanding patient care access to medication management, chronic disease management, and care for non-emergency acute conditions. However, realizing this potential requires a community pharmacy practice model that can sustainably support the provision of patient care services.

### The community pharmacy payment model

Despite expanded clinical training and scope of practice, community pharmacy operations remain largely dependent on prescription dispensing rather than provision of patient care services.^20,35^ This creates a fundamental disconnect between how community pharmacies are paid and the care pharmacists are trained and authorized to provide and that patients and communities increasingly need.^36,37^ Community pharmacy revenue remains largely tied to prescription dispensing, while reimbursement for pharmacist-provided patient care services remains inconsistent and limited.^20,38,39^ As a result, pharmacists are often not reimbursed for their expert assessment of medication appropriateness, including ensuring a clear indication for therapy, evaluating effectiveness in achieving clinical goals, assessing safety to minimize adverse effects and interactions, and supporting patient adherence to optimize outcomes.

This dispensing-based payment model requires a high prescription volume, diverse payor mix, lean staff, and rapid turn-around to remain financially viable.^40^ This model makes it difficult for community pharmacies to dedicate the pharmacist time, staff, and infrastructure needed to provide patient care services.^41^ Therefore, expanded pharmacist training and scope of practice alone are not sufficient to support these services. Sustainable delivery of these services also requires mechanisms to reimburse community pharmacists for the care they provide.

The consequences of the current payment model extend beyond the ability of pharmacists to provide patient care services. Many communities are experiencing a growing number of pharmacy closures, particularly in rural and underserved areas.^42^ These closures contribute to the emergence of “pharmacy deserts,” leaving communities with reduced access to medications and pharmacist care.^43^ Rural areas and low-income and underrepresented communities are disproportionately affected, potentially exacerbating existing geographic and socioeconomic disparities in access to care.^42,43^ Pharmacy deserts are also linked to lower medication adherence, worse chronic disease management, higher healthcare costs, and increased hospitalization rates.^44^

The sustainability of community pharmacy access is closely tied to reimbursement and policies that shape the financial viability of pharmacy practice. Payment policies not only influence whether community pharmacists are reimbursed for patient care services, but their design can also influence whether those services can be implemented and scaled in practice. In the following sections, we examine existing and proposed state and federal payment policies, with particular attention to how differences in payer applicability, patient and service eligibility, and alignment with state-authorized scope of practice influence implementation and scalability. We then identify opportunities to strengthen payment policy and address additional implementation needs, including health information exchange and credentialing.

## Current payment policy landscape

### State policy

Over the past decade, states have led much of the progress toward payment for pharmacist patient care services.^38,39^ As of 2025, 43 states have had regulatory or policy changes that recognize pharmacists as health care providers and require state Medicaid plans or state-regulated commercial plans to reimburse pharmacists for covered services.^39^ Rising health care costs and state responsibility for Medicaid budgets have motivated legislators across party lines to seek cost-saving solutions. Pharmacists have demonstrated their ability to lower overall healthcare costs across a variety of chronic conditions, and their expanded role has been recommended for over a decade by both the US Public Health Services and National Governors Association.^29,45^ The COVID-19 pandemic further highlighted community pharmacists' accessibility and public trust through testing and vaccination services as well as collaboration with other health professionals.^46^

Despite this momentum, state policies vary widely in scope, payer applicability, and operational details. Of the 43 states with regulatory or policy changes, 17 require commercial insurers to provide broad payment parity, reimbursing pharmacists for any services within their scope that are covered when provided by other health care professionals.^39^ Six additional states offer more limited payment parity with commercial insurers, covering only a narrow set of services. Medicaid coverage requirements for pharmacist services have been broadly established in 22 states, although these policies vary considerably in scope and implementation requirements, while an additional 19 states have mandated coverage for a narrower set of services. Further, in many states, pharmacists must also follow specific protocols, participate in collaborative practice agreements, or hold specific credentials to qualify for reimbursement.

These differences in payer applicability, covered services, and reimbursement requirements have important implications for implementation. Significant variation from state to state makes implementation difficult to operationalize in pharmacies with locations across multiple states. Differences in coverage across payers or patient populations can result in some patients being eligible for pharmacist-provided care while others receiving care at the same pharmacy are not. This fragmentation complicates workflow standardization, staff training, infrastructure investment, and clear communication about available pharmacist services. In some cases, restrictive eligibility criteria may leave pharmacies with too few eligible patients to make a service financially viable, limiting the operational consistency needed to sustain community pharmacist services.

### Federal policy

The Pharmacy and Medically Underserved Areas Enhancement Act was first introduced in 2014 (H.R. 4190) and was most recently brought to the 119 Congress in September 2025 (S.2800).^47,48^ This bill authorizes pharmacist reimbursement under Medicare Part B for services delivered in designated shortage areas, provided those services would otherwise qualify for payment if performed by a physician.

A key limitation of this legislation is its narrow patient eligibility criteria. It restricts pharmacist reimbursement to patients in medically underserved areas (MUAs) or populations (MUPs) and health professional shortage areas (HPSAs).^49^ These restrictions were intended to reduce the bill's fiscal impact and prioritize areas already recognized as needing more healthcare professionals. A 2021 analysis found that only 56% of pharmacies nationwide were located in MUAs/MUPs or HPSAs, with substantial variation across states.^49^ This finding suggests that a geographically restricted reimbursement framework may exclude many pharmacies capable of expanding access to care within their communities. Further, this variation in eligibility would likely create implementation challenges at the practice level, especially for organizations operating multiple pharmacy locations, as it complicates consistent delivery of pharmacist services and limits their potential to improve patient access, health outcomes, and cost savings.^50^

Introduced in response to the COVID-19 pandemic, legislation now known as the Main Street Pharmacy Access Act was first introduced in 2022 as the Equitable Community Access to Pharmacist Services (ECAPS) Act (H.R. 7213) and most recently reintroduced in the 119^th^ Congress in May 2025 (H.R. 3164/S.242).^51-53^ The bill authorizes pharmacist reimbursement via Medicare Part B, when acting within their state-authorized scope of practice, to evaluate and treat patients for COVID-19, influenza, respiratory syncytial virus (RSV), streptococcal pharyngitis, or other services addressing declared public health emergencies. A notable strength of this bill is its deferral to state-level definitions of pharmacists' scope of practice, supporting payment for services legally authorized in their state. Like the Pharmacy and Medically Underserved Areas Enhancement Act, the Main Street Pharmacy Access Act restricts patient eligibility to a limited number of specific services. The bill does not allow for broad access to pharmacist care across diverse patient populations or enable consistent, large-scale implementation of pharmacist services, particularly for chronic medical conditions. As a result, the Main Street Pharmacy Access Act functions more as a targeted public health response than a durable framework for comprehensive pharmacist integration into longitudinal patient care.

Collectively, current state and federal policies demonstrate growing recognition of pharmacists as patient care providers, yet 3 design features consistently influence implementation and scalability: payer applicability, patient and service eligibility, and alignment with state-authorized scope of practice. These findings point to 3 areas for future policy design and implementation.

## Payment policy reforms

### Broad payer coverage

Several state-level reforms already offer useful templates for broad payer coverage, demonstrating that pharmacist medical billing is both operationally feasible and politically attainable in diverse policy environments. Successful state reforms include policies that require Medicaid and state-regulated commercial plans to recognize pharmacists as providers and reimburse them for clinical services within their scope of practice and explicit language prohibiting compensation discrimination when the same service would be covered if furnished by another licensed professional.^38^

At the national level, broad payer coverage for immunization services provides another model of how reimbursement for community pharmacy care can provide significant benefits.^54^ Interoperability between pharmacy dispensing systems and state immunization information systems (IIS), as well as broad payer coverage, were key facilitators of this impact. For example, 3 projects across Nebraska, Iowa, Pennsylvania, and Washington demonstrated a 15%-33% increase in adult influenza, pneumococcal, herpes zoster, and pertussis vaccinations across more than 300 community pharmacies utilizing IIS connectivity and proactive vaccine recommendation.^55-57^ Broad payer coverage also plays a central role, as dual medical–pharmacy benefit designs are associated with higher adult vaccination rates and pharmacies experience exceptionally high claim reimbursement, reducing financial barriers for both patients and providers.^58,59^ Together, these lessons from successful community pharmacy-based vaccination programs highlight that aligning coverage, reimbursement reliability, and clinical data connectivity creates the conditions necessary for community pharmacies to consistently deliver timely, accessible patient care and offers a policy framework to support broader patient care services.

Further, pharmacists practicing in federal health facilities, like the Veterans Affairs and Indian Health Service, have long provided broad clinical services. An analysis of the federal government's extensive performance data has demonstrated that federally funded patient care services delivered by pharmacists can improve patient outcomes, promote patient involvement, and reduce demands affecting the health care system.^29^ Notably, these benefits to patients and the healthcare systems were accompanied by significant benefit-to-cost for each dollar invested in the clinical pharmacy service. The overall average benefit gained was $10.07 per $1 of allocated funds. Together, these state and federal experiences provide evidence for broadly supporting reimbursement for pharmacist-provided care across payers.

### Broaden patient and service eligibility

Future policy should build on emerging state and national models by preserving their core structure of paying pharmacists under the medical benefit for services that are already covered when delivered by other professionals. Eligibility should be broadened beyond specific geographies or narrowly defined conditions and align Medicare, Medicaid, and commercial coverage to reduce administrative complexity. Experience with state policy variation and recent federal bills shows that restrictive eligibility criteria, such as limiting reimbursement to patients in MUAs/MUPs or HPSAs or to a narrow set of clinical scenarios, may reduce short-term fiscal exposure but create substantial practice-level barriers. Current federal legislation demonstrates congressional willingness to recognize pharmacists under Part B but remains incomplete, leaving major gaps in patient eligibility, service scope, and long-term sustainability.

The most direct and comprehensive approach to ensuring patient access to pharmacist services is to amend Section 1861(s) of the Social Security Act to recognize pharmacists as practitioners eligible for Part B payment for covered services within their scope of practice, not just in shortage areas. Reimbursement should be comparable to that of other providers, taking into account the level of service provided, standard credentialing practices, and established reimbursement mechanisms that already exist. Patient access should not be restricted by geography or community characteristics; statutory language that ties coverage to specific zip codes, counties, or census tracts should be avoided because it impedes implementation when pharmacies serve mixed populations or when patients travel across boundaries. Instead of hard eligibility exclusions, better policy should give the Centers for Medicare & Medicaid Services (CMS) flexibility to prioritize high-need populations through risk-adjusted payment or value-based models while maintaining broad baseline eligibility for pharmacist services. These efforts will allow pharmacists to help fill the primary care shortage needs of a particular area where they practice rather than be policy limitations that are not helping the general population where they live.

### Defer scope of practice to the states

Scope of practice for pharmacists has historically been regulated at the state level, and recent expansions have occurred primarily through state legislatures and boards of pharmacy. Payment policy at all levels should continue to defer to state-authorized scope of practice, allowing reimbursement to evolve as states expand pharmacists' patient care authority without requiring separate changes to payment policy for each service. Both the Pharmacy and Medically Underserved Areas Enhancement Act and the Main Street Pharmacy Access Act explicitly condition Medicare payment on services being delivered within the pharmacist's state-authorized scope of practice. Requiring CMS to accept state-documented scope of practice changes without additional federal rulemaking for each specific service will enable timely adaptation as states modernize their laws. Preserving and generalizing this approach allows states to continue to innovate, tailoring pharmacist authorities to local needs, workforce capacity, and health system priorities while simplifying federal implementation by linking covered pharmacist services to existing state law and regulation.

## Additional implementation considerations

Community pharmacists face additional barriers to implementing services and receiving reimbursement, demonstrating that even well-designed payment reform is not sufficient to support sustainable pharmacist-provided care.^60^ Additional policy and regulatory reforms are needed to address barriers that can prevent community pharmacists from implementing patient care services even when reimbursement is available. In particular, health information exchange and credentialing represent 2 important implementation considerations that must be addressed alongside payment form.

### Expand health information exchange to support team-based care

Community pharmacists' ability to deliver high-quality chronic disease management, medication optimization, and acute care services depends on timely access to relevant clinical information (eg, diagnoses, laboratory values, problem lists) and on their capacity to document interventions in a way that is visible to other members of the care team. Payers contracting with pharmacists for medical-benefit services must support interoperable electronic documentation and information exchange, consistent with federal information-blocking rules and health information technology (IT) standards.^61,62^

Policy and regulatory reforms should promote bidirectional health information exchange by incentivizing or requiring payors and health systems to extend access to community pharmacists who are participating as medical-benefit providers. They should also strengthen interoperability and participation in health information exchange by aligning pharmacist participation with federal interoperability initiatives so that pharmacy systems can exchange structured clinical data (eg, lab results, care plans, medication changes) with physician practices, health systems, and other providers, and by supporting technical assistance and funding to help community pharmacies, particularly small and independently owned sites, implement or upgrade health IT systems to meet these requirements.

### Standardize credentialing pathways for pharmacists

Credentialing with health plans can be challenging for pharmacists because they represent a relatively new provider type and the infrastructure for documenting and verifying their qualifications remains fragmented and underdeveloped.^63,64^ These barriers can delay or discourage implementation even when payment policies formally authorize reimbursement.^65^ In one survey of pharmacists, there was widespread dissatisfaction with existing credentialing systems, driven in part by poor interoperability between credentialing platforms, redundant data entry requirements, and data security concerns, creating unnecessary administrative burden and slowing adoption.^66^ Implementation barriers extend beyond infrastructure to include significant time, cost, and variability in credentialing expectations across organizations, practice settings, and health plans.^67^

Health plans and payers should take a more proactive role in standardizing and modernizing credentialing pathways for pharmacists to support the sustainability of pharmacist-provided care. Payers should develop standardized credentialing pathways specific to pharmacists, invest in centralized or interoperable credentialing systems, and align credentialing policies with reimbursement models that reflect pharmacists' clinical contributions.

## Conclusion

Increasing health care demands and provider shortages in the US call for additional models of care, with community pharmacists well positioned to help meet patients' needs for medication management, chronic disease management, and timely access to non-emergency acute care. However, realizing this potential requires policy reforms that remove barriers preventing community pharmacies from transitioning from a product-centric, volume-driven model to a sustainable, service-oriented, patient-centered model that supports patients' health needs, promotes safe and effective medication use, and helps reduce healthcare expenditures. Payment policies should establish broad medical benefit coverage across payers, avoid restrictive patient and service eligibility criteria, and defer scope of practice decisions to the states. Additional policy and implementation reforms are needed to address implementation barriers related to electronic health record integration and credentialing. Together, these reforms can support sustainable and scalable community pharmacist-provided care and improve access, quality, and affordability of care.

## Supplementary Material

qxag252_Supplementary_Data
