# Supplementary material for: Building on the 2025 national academies pharmacy workshop: ensuring access to community pharmacist care
Source: Health Aff Sch. 2026 Sep 18;4(10):qxag252. doi: 10.1093/haschl/qxag252 (PMC13682904; doi:10.1093/haschl/qxag252)
Supplement: Supplementary file 1 [file qxag252_supplementary_data.zip › Author Disclosure_Melin.docx]

| ICMJE DISCLOSURE FORM | |
| --- | --- |
| **Date:** | 8/24/2026 |
| **Your Name:** | Kyle Melin |
| **Manuscript Title:** | Building on the 2025 National Academies Pharmacy Workshop: Ensuring Access to Community Pharmacist Care |
| **Manuscript Number (if known):** | HASCHOLAR-D-26-00342 |
| In the interest of transparency, we ask you to disclose all relationships/activities/interests listed below that are related to the content of your manuscript. “Related” means any relation with for-profit or not-for-profit third parties whose interests may be affected by the content of the manuscript. Disclosure represents a commitment to transparency and does not necessarily indicate a bias. If you are in doubt about whether to list a relationship/activity/interest, it is preferable that you do so.  The author’s relationships/activities/interests should be defined broadly. For example, if your manuscript pertains to the epidemiology of hypertension, you should declare all relationships with manufacturers of antihypertensive medication, even if that medication is not mentioned in the manuscript.  In item #1 below, report all support for the work reported in this manuscript without time limit. For all other items, the time frame for disclosure is the past 36 months. | |

|  | | | **Name all entities with whom you have this relationship or indicate none (add rows as needed)** | **Specifications/Comments (e.g., if payments were made to you or to your institution)** |
| --- | --- | --- | --- | --- |
| **Time frame: Since the initial planning of the work** | | | | |
| **1** | All support for the present manuscript (e.g., funding, provision of study materials, medical writing, article processing charges, etc.)  **No time limit for this item.** | | ☒ \| **None** \| Click the tab key to add additional rows. | |
| **Time frame: past 36 months** | | | | |
| **2** | | Grants or contracts from any entity (if not indicated in item #1 above). | ☐ \| **None** NIH/NIGMS (Grant 5U54GM133807) \| Director of Collaborative Projects Program; NIH and the National Alliance for Hispanic Health (Grant 3OT2OD025277) \| Sub-award to support outreach for All of Us Research Program; NIH/NIGMS (Grant 2R25GM116740) \| Sub-award to provide mentorship and instruction to early-career researchers participating in the LEADS program; NIH/NIGMS (Grant 5R16GM149372) \| Key-personnel on clinical research project.; CDC and the National Alliance for Hispanic Health (Grant NH23IP922642) \| Sub-award to support community-based immunization efforts; Healthy Americas Foundation \| Site director for Vive tu Vida! Get Up, Get Moving! Community health fair | |
| **3** | | Royalties or licenses | ☒ \| **None** | |
| **4** | | Consulting fees | ☐ \| **None** Healthy Americas Foundation \| Consultant on the Healthy Americas Research Consortium; Pharmpix Corportation \| P&T Committee Member | |
| **5** | | Payment or honoraria for lectures, presentations, speakers bureaus, manuscript writing or educational events | ☐ \| **None** Specialty Pharmaceutical Summit \| Invited panelist on Alternate Coverage Mechanisms for Rare Disease Therapeutics; Mid-Atlantic AIDS Education and Training Center (MAAETC) \| Various education sessions for healthcare professionals on HIV care; American College of Cardiology Puerto Rico Chapter \| Educational sessions for allied health professionals; Colegio de Farmaceutics de Puerto Rico \| Educational session on AI for pharmacists; Puerto Rico Science, Technology And Research Trust \| Educational sessions on opioid use disorder | |
| **6** | | Payment for expert testimony | ☒ \| **None** | |
| **7** | | Support for attending meetings and/or travel | ☐ \| **None** American Pharmacists Association \| Support provided as an Associate Editor of the *Journal of the American Pharmacists Association* to participate in annual editors meeting | |
| **8** | | Patents planned, issued or pending | ☒ \| **None** | |
| **9** | | Participation on a Data Safety Monitoring Board or Advisory Board | ☒ \| **None** | |
| **10** | | Leadership or fiduciary role in other board, society, committee or advocacy group, paid or unpaid | ☒ \| **None** | |
| **11** | | Stock or stock options | ☒ \| **None** | |
| **12** | | Receipt of equipment, materials, drugs, medical writing, gifts or other services | ☒ \| **None** | |
| **13** | | Other financial or non-financial interests | ☒ \| **None** | |
|  | |  |  | |
| **Please place an “X” next to the following statement to indicate your agreement:** | | | | |
| ☒ | | I certify that I have answered every question and have not altered the wording of any of the questions on this form. | | |
