# Supplementary material for: Building on the 2025 national academies pharmacy workshop: ensuring access to community pharmacist care
Source: Health Aff Sch. 2026 Sep 18;4(10):qxag252. doi: 10.1093/haschl/qxag252 (PMC13682904; doi:10.1093/haschl/qxag252)
Supplement: Supplementary file 1 [file qxag252_supplementary_data.zip › Author Disclosure_Bacci.docx]

| ICMJE DISCLOSURE FORM | |
| --- | --- |
| **Date:** | 8/24/2026 |
| **Your Name:** | Jennifer Bacci |
| **Manuscript Title:** | Building on the 2025 National Academies Pharmacy Workshop: Ensuring Access to Community Pharmacist Care |
| **Manuscript Number (if known):** | HASCHOLAR-D-26-00342 |
| In the interest of transparency, we ask you to disclose all relationships/activities/interests listed below that are related to the content of your manuscript. “Related” means any relation with for-profit or not-for-profit third parties whose interests may be affected by the content of the manuscript. Disclosure represents a commitment to transparency and does not necessarily indicate a bias. If you are in doubt about whether to list a relationship/activity/interest, it is preferable that you do so.  The author’s relationships/activities/interests should be defined broadly. For example, if your manuscript pertains to the epidemiology of hypertension, you should declare all relationships with manufacturers of antihypertensive medication, even if that medication is not mentioned in the manuscript.  In item #1 below, report all support for the work reported in this manuscript without time limit. For all other items, the time frame for disclosure is the past 36 months. | |

|  | | | **Name all entities with whom you have this relationship or indicate none (add rows as needed)** | **Specifications/Comments (e.g., if payments were made to you or to your institution)** |
| --- | --- | --- | --- | --- |
| **Time frame: Since the initial planning of the work** | | | | |
| **1** | All support for the present manuscript (e.g., funding, provision of study materials, medical writing, article processing charges, etc.)  **No time limit for this item.** | | ☐ \| **None** National Academies of Medicine (NAM) \| Payment made to institution as part of the NAM Fellowship in Pharmacy to support the fellow’s professional and academic career; \| Click the tab key to add additional rows. | |
| **Time frame: past 36 months** | | | | |
| **2** | | Grants or contracts from any entity (if not indicated in item #1 above). | ☐ \| **None** National Association of Chain Drug Stores Foundation \| Payment to institution to contribute expertise to Foundation’s Faculty Scholars Program; Merck & Co. \| Payment to institution to support research related to community pharmacy-based HPV vaccine delivery; Community Pharmacy Foundation \| Payment to institution to support research on community pharmacy-based care for opioid use disorder; UCB Pharma \| Payment to institution to support research on community pharmacy-based epilepsy care | |
| **3** | | Royalties or licenses | ☒ \| **None** | |
| **4** | | Consulting fees | ☒ \| **None** | |
| **5** | | Payment or honoraria for lectures, presentations, speakers bureaus, manuscript writing or educational events | ☐ \| **None** American Association of Colleges of Pharmacy (AACP) \| Honoraria to present at national virtual workshops on community pharmacy practice and medical billing; Paid to individual | |
| **6** | | Payment for expert testimony | ☒ \| **None** | |
| **7** | | Support for attending meetings and/or travel | ☐ \| **None** American Association of Colleges of Pharmacy (AACP) \| Support to attend the 2025 AACP Annual Meeting and facilitate the Science Plenary session; Paid to individual; National Academies of Medicine (NAM) \| Support to attend NAM Annual Meetings, Emerging Leader Forums, and other fellowship activities during the 2024 - 2026 NAM Fellowship in Pharmacy; Paid to individual | |
| **8** | | Patents planned, issued or pending | ☒ \| **None** | |
| **9** | | Participation on a Data Safety Monitoring Board or Advisory Board | ☒ \| **None** | |
| **10** | | Leadership or fiduciary role in other board, society, committee or advocacy group, paid or unpaid | ☐ \| **None** Washington State Pharmacy Association \| No payment to individual or institution; Volunteer role | |
| **11** | | Stock or stock options | ☒ \| **None** | |
| **12** | | Receipt of equipment, materials, drugs, medical writing, gifts or other services | ☒ \| **None** | |
| **13** | | Other financial or non-financial interests | ☒ \| **None** | |
|  | |  |  | |
| **Please place an “X” next to the following statement to indicate your agreement:** | | | | |
| ☒ | | I certify that I have answered every question and have not altered the wording of any of the questions on this form. | | |
